# Issues in Identifying Strategies for Youth Mental Well-Being in Stockholm Municipalities Using Participatory Sessions and Text Mining: Qualitative Study

**DOI:** 10.2196/66377

**Published:** 2025-07-28

**Authors:** Harsha Krishna, Adam S Darwich, Sebastiaan Meijer

**Affiliations:** 1 Division of Health Informatics and Logistics, KTH Royal Institute of Technology, Hälsovägen 11C, Huddinge, Stockholm, 141 57, Sweden, 46 8 790 60 00

**Keywords:** youth, mental wellbeing, Stockholm, participatory workshop, data driven, visualizations, boundary objects, text mining, municipalities

## Abstract

**Background:**

Socioeconomic and environmental factors influence youth mental well-being. Promoting mental well-being is essential to support youths’ development toward adulthood with good mental health. Different Stockholm municipalities have adopted strategies to promote youth well-being. However, contextualizing and perceiving goals and mechanisms at the local municipal level is difficult. Thus, comparing or tracking their conception, purpose, and characteristics has been challenging.

**Objective:**

We aimed to use data visualizations developed from a fusion of data sources to facilitate stakeholder conversations on promoting youth mental well-being within a municipality. We strive to demonstrate our methodology of using data visualizations as “boundary objects,” which are cognitive artifacts that bridge knowledge from various domains to elicit understanding from specialized and siloed parts of a health delivery system.

**Methods:**

Stakeholders from the municipalities of Lidingö and Nynäshamn participated in the study. A total of 15 workshops were conducted: 6 with only Lidingö participants, 6 with only Nynäshamn participants, and 3 with mixed participants. The sessions were conducted via Microsoft Teams or as physical sessions in Swedish and lasted between 60 and 90 minutes. Interactions were recorded with consent from participants. Recordings were transcribed using Amberscript software. We used matrix factorization with Kullback–Leibler divergence to extract 1000 features and created 10 topic clusters with 20 top words. We used the identified words and phrases to backtrack within the transcripts and to identify dialogues where they were used. We summarized participants’ interactions across all the workshops to identify factors or strategies discussed for youth well-being.

**Results:**

Participants noted that these sessions allowed them to contextualize their local observations from municipalities relative to the status of other municipalities in the national statistics. They indicated that they conceptualized well-being differently in their respective municipalities and between different professional backgrounds, and the sources of stress for youth differed. They noted the differences in the strategy and data collected for tracking youth well-being. Promotion of sports was a common strategy, while options for leisure activities differed between municipalities and professions.

**Conclusions:**

Based on our observations and analysis of the transcripts from participatory workshops, we observed that the data-driven visualizations helped stakeholders from different departments of Lidingö and Nynäshamn municipalities to identify and bridge knowledge gaps caused by data silos. Participants noted proposals to modify future surveys and identified that this approach to visualizations would help them to share knowledge and maintain a long-term and sustainable collaboration across departments.

## Introduction

Sweden follows the World Health Organization (WHO) and Organisation of Economic Co-operation and Development (OECD) definition of good mental well-being as “a state of well-being in which the individual realizes his or her abilities, can cope with the normal stresses of life, can work productively and fruitfully, and can make a contribution to his or her community” [1-3]. The definition uses the 2*-*continuum model identified by Keyes et al [4], which states that individuals can be classified based on their mental illness and mental health status. According to this classification, a person with high mental health and low mental illness is stated to be “flourishing,” and the contrary is said to be languishing. A person with high mental health but enduring mental illness is considered flourishing with mental illness. It demonstrates that there are different factors, both genetic and environmental, that contribute to an individual’s well-being. It also argues that despite enduring mental illness, an individual can lead a meaningful life and flourish. Targeting individuals when they are young ensures a preventative approach to well-being in the future by equipping them with strategies to manage and seek help early. Based on this goal, the Region of Stockholm adopted a goal to improve the mental well-being of its youth population.

**Figure 1.**
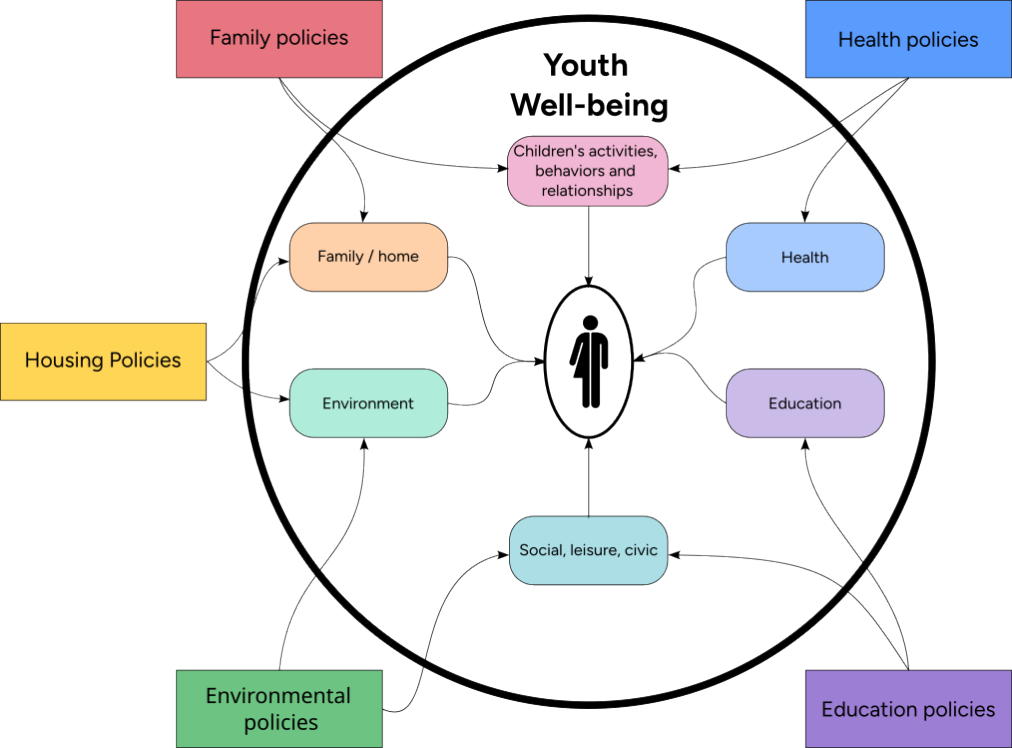
Parameters involved in determining youth well-being, adapted from the Organisation for Economic Co-operation and Development [2].

Figure 1 lists some factors that potentially affect an individual’s well-being, including environmental and physiological aspects, such as access to education, leisure time, and so on. These factors are influenced by various programs and policies implemented and monitored by local municipalities, as described in previous studies [2-8]. The work done by Moustaid et al [6] modeled the factors, such as education, social environments, schools, labor markets, and health care provisions, that affect mental health in cities. They demonstrated that poor mental health in cities could result from poor coordination between different city infrastructures. This was explored using a systems dynamics–based model in a participatory approach with other stakeholders.

Typically, different departments in a municipality would be responsible for monitoring or implementing domain-specific strategies. This has led to data silos and myopic perspectives within departments in the municipal system. Well-being promotion strategies tend to cut across various departmental responsibilities. Therefore, a participatory session would bring this siloed data together and make it understandable for all domains.

**Figure 2.**
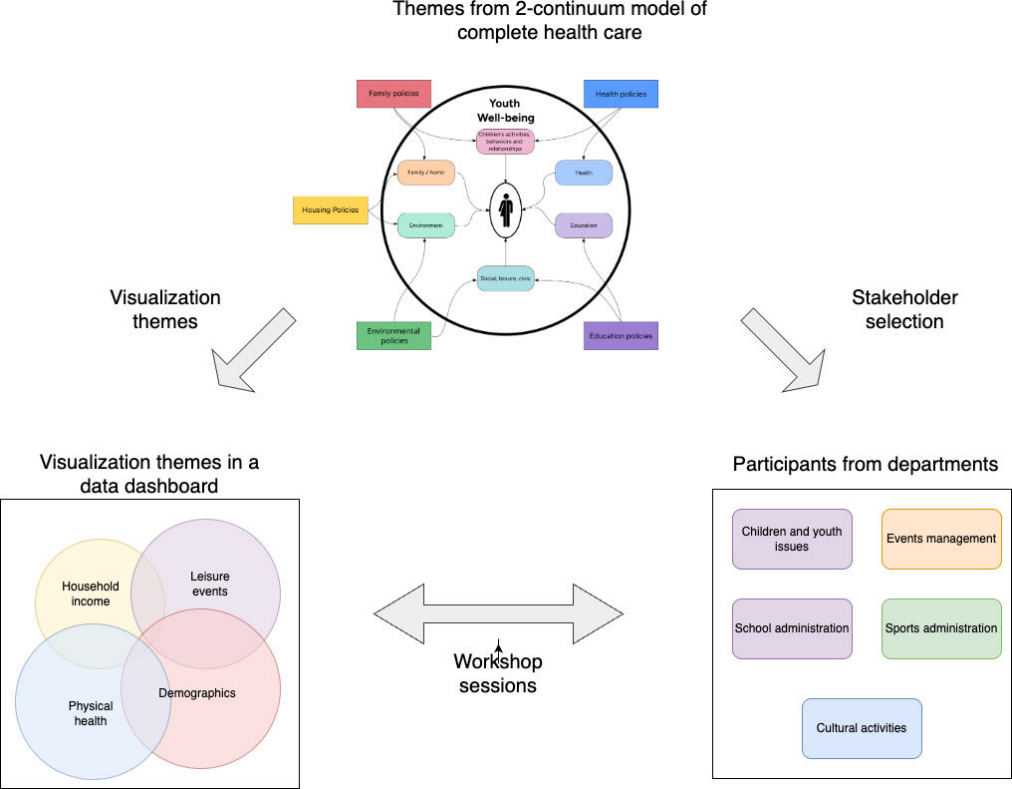
Well-being–related data and its organization into silos between departments.

As Figure 2 shows, no shared data infrastructure monitors all the identified dimensions. We used a fused dataset, which combined all the data available in a network database, to query across them.

To help understand the goals and perspectives at the municipal level, we propose to use visualizations generated from datasets that influence mental health as “boundary objects.” Boundary objects are defined as objects that live in multiple social worlds, each with a different identity. For example, an engineering drawing acts as a boundary object in manufacturing processes. It allows designers and fabricators to discuss challenges in their departments while working toward the same objective. Boundary objects and the theories of knowledge boundaries have been used to help work with complex interconnected systems, as described in previous studies [9-12], to further demonstrate and develop the theory.

Boundary objects help contextualize knowledge when working with expert stakeholders from different domains. It was proposed in the seminal paper [10]. The authors describe boundary objects as plastic enough to adapt to the local needs and constraints of several parties using them, yet robust enough to maintain a common identity across sites. Thus, they satisfy the informational requirements of various communities while still having distinct meanings in each of them. Boundary objects emerge through work processes. Boundaries refer to the overlap of areas between different communities.

**Figure 3.**
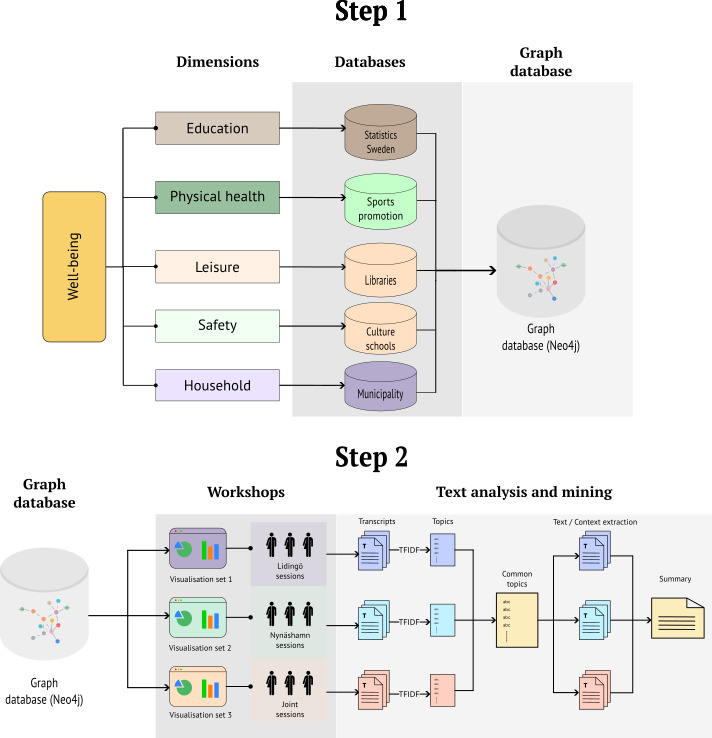
Overview of the complete methodology.

We propose using boundary objects to collaborate with different municipal departments. Data-driven visualizations can be used as boundary objects in participatory sessions, enabling different experts to discuss youth mental well-being. Authors in [13-15] have used visualizations as boundaries for building models in group sessions. Figure 3 illustrates an approach for using visualizations in a workshop. In the first step, a fused dataset is developed using data from national statistics and data from different municipal departments.

In the second step, various visualizations are generated based on different themes. The visualizations can be used within a workshop to discuss participants’ experiences concerning various well-being promotion programs.

We built a fused dataset from separate sources to explore the different promotion programs and their reasons. We created it using a network database, Neo4j [16], which we explained in detail in our previous work. We used the fused dataset to build cross-sectoral visualizations, which we used in workshop sessions with participants from 2 municipalities, including the youth.

## Methods

### Overview

**Figure 4.**
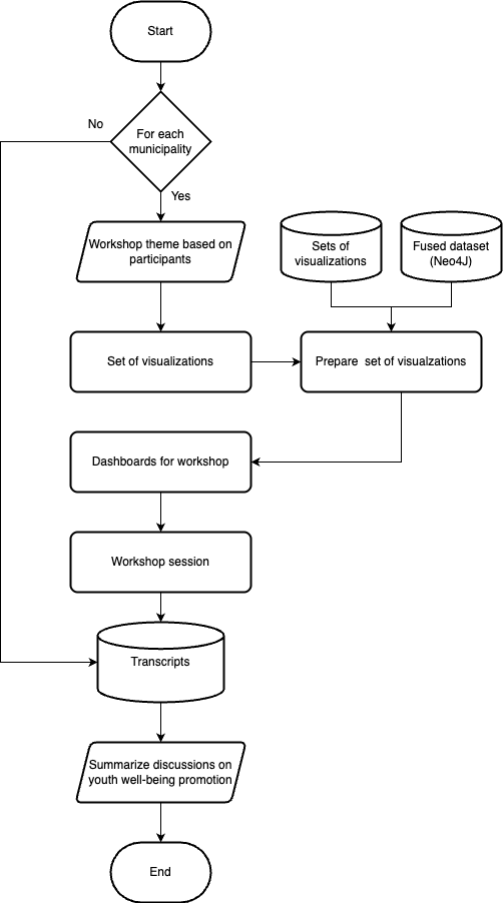
Research methodology.

Figure 4 illustrates the research method. Two municipalities, Lidingö and Nynäshamn, agreed to collaborate on the project after a general request to all the municipalities of Region Stockholm. We collected data from national statistics [17] and different departments of Lidingö and Nynäshamn. We also received data from social services, schools, and other surveys run by the municipalities with their youth population.

For our study, we studied the population aged between 16 and 29 years. This was based on initial research and discussions with the municipalities. We visualized data from 2018-2019 and 2022-2023 to demonstrate changes observed in the data collected from youth between 19 and 29 years, split by sex. We note that the data from 2020 and 2021 would have recorded the impact of the global COVID-19 pandemic. While Sweden did not implement a complete lockdown, education was shifted to an online format, which could have caused variations in mental health during that period.

For each municipality, we developed a set of different visualizations based on the factors identified for youth well-being, as shown in Figure 1, and based on the various participants for a given workshop session.

We created workshop assets, which included (1) an introductory presentation, (2) a set of data visualizations, (3) a video summary of the data being presented and access to the data dashboard, and (4) a set of discussion topics for the workshop.

A fused database allowed us to query data across different themes. For example, we could retrieve data on the education, environment, home condition, and neighborhood safety of a given age group of youth in a municipality from the same database.

The visualizations included bar graphs, pie charts, and line graphs. We provided data sources with periods, local or national, and the name of the study, along with the visualizations. Multiple visualizations were arranged on different pages based on education, leisure activities, sports, and so on.

As per the definition of a boundary object, each dashboard was created with visualizations of data from national statistics and data from the participants’ departments. The selections were based on (1) the municipality that participants belong to and participants’ expertise who are attending the workshop.

For example, for a session with Lidingö, if the participants were from sports organizations and clubs along with the municipal administrators and educators, a dashboard was constructed by combining data from sports organizations about youth between the ages of 15‐25 years for the selected time period of 2019‐2023 in Lidingö. Data on school attendance and academic performance, national statistics, and demographics of the youth in Lidingö would also be added and visualized. If any further programs or campaigns were organized, their performance was also added to the dashboard. This presented an overview of the leisure, education, and background of the youth population from similarly grouped populations.

**Figure 5.**
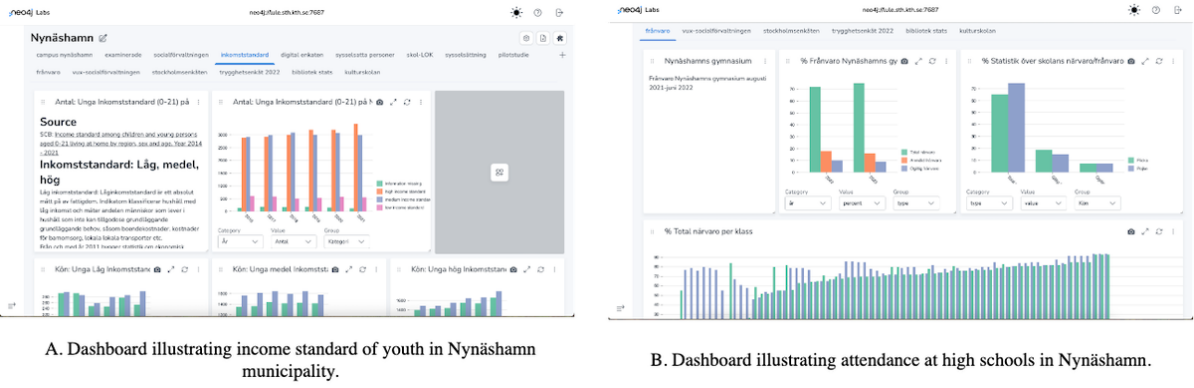
Dashboard illustrating income standard and high school attendance of youth in Nynäshamn municipality.

Figure 5 illustrates an example of data visualizations developed on the Neo Dash platform (Neo4j Labs) [18] with the Neo4j graph database.

It demonstrates the income standard visualization for Nynäshamn municipality, which is targeted at policy makers and school personnel sessions. This would be combined with the visualization of school attendance and the activities arranged in Nynäshamn by the libraries, which target the youth. This allowed us to change and compose different pages and visualization sets based on the participants’ backgrounds for a workshop. It also allowed us to construct new visualizations on demand during a workshop.

**Figure 6.**
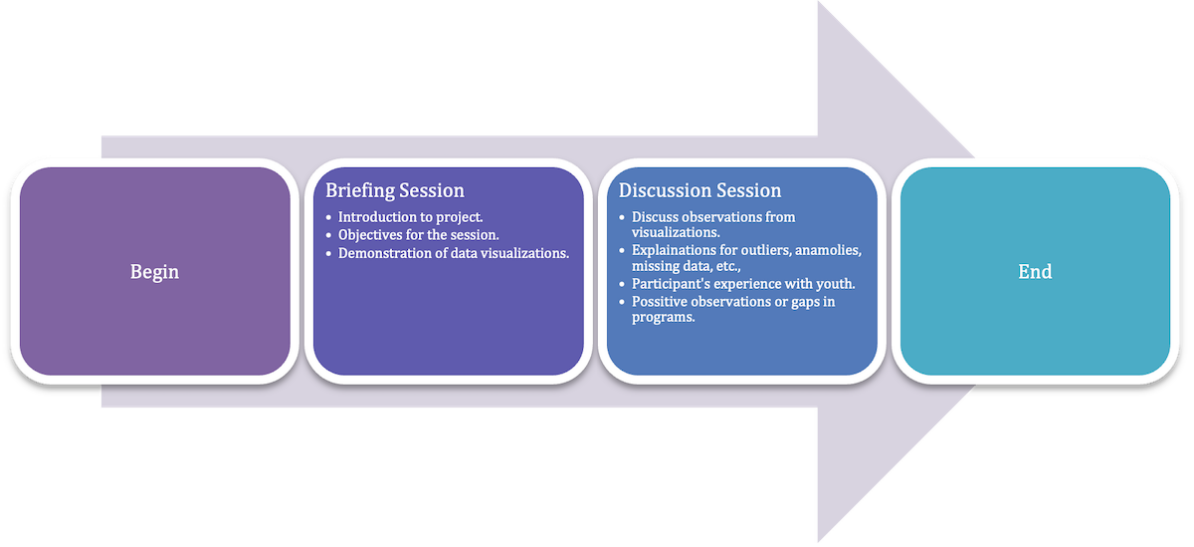
Conducting a workshop session.

We began each workshop with a briefing followed by a discussion. Figure 6 illustrates the workshop’s progress and various steps. The workshops were scheduled between 60 and 90 minutes. A screenshot from one such session is included in the Multimedia Appendices (Figure S1 in Multimedia Appendix 1).

All workshops were conducted via Microsoft Teams except the first one with Lidingö, which was held as a physical session. The appendices list all workshops conducted, their themes, and participation details (Table S2 in Multimedia Appendix 2).

Participants’ consent for recording was obtained for both online and physical sessions. Notes and audio were collected during all sessions. Online sessions were recorded using conference software and transcribed using the Amberscript software [19]. All the content and analysis were in Swedish. We have included the content in both Swedish and English in the Multimedia Appendices.

We used the minibatch nonnegative matrix factorization with the Kullback–Leibler divergence [20,21] to analyze the content and identify the most critical topics. We varied the n-grams, that is, the length of phrases, between 2 and 3 words to identify the top words and phrases used in each text corpus under consideration. We developed Python scripts with scikit-learn libraries [22-25]. The text was processed by removing the timestamps from the transcripts and creating a stop-word list to avoid identifying pronouns, numbers, and member names. A list of all the Swedish stop-words identified for processing is provided in the Multimedia Appendices (Table S3 in Multimedia Appendix 3).

We set the system to sample the corpus for 2000 samples and extract at least 1000 features. We then extracted at least 10 topics or clusters with 20 top words in batches of 128. The algorithm extracted the top 20 clustered words and formed 10 such clusters. Table S1 in Multimedia Appendix 4 lists the top words identified in Swedish. We traced the identified words back to the transcripts to identify and summarize the context of the discussions (MultimediaAppendices 5  6). We summarize the discussions as follows:

1. How participants approached defining well-being.
2. Tracking youth well-being with currently available data.
3. How leisure time is used by youth.
4. Current strategies to improve youth well-being.

### Ethical Considerations

Verbal consent was obtained from all participants at the recording of each workshop session for analysis and research purposes. No identifiable information has been used in the analysis apart from the target municipality for the sessions. Participants could leave any of the sessions at any time before the recording began. All analysis is conducted without specific names. The data used for visualizations are from publicly available sources from Statistics Sweden.

## Results

The top 10 topics from the corpus with the Lidingö municipality workshop sessions are illustrated in Figure S2 in Multimedia Appendix 7. Similarly, the topics selected from the corpus of Nynäshamn and the joint workshop sessions are illustrated in Figures S3 and S4 in Multimedia Appendix 3 and MultimediaAppendices 8  9, respectively. The graphs represent the numerical score of the words as a combination of 2 to 3 words per the n-grams setting. Each cluster represents a topic cluster internally arranged according to the calculated score. The score is calculated using the ratio of term frequency, the number of times the term occurs in a document, and inverse document frequency, the inverse number of documents in which the term appears. A matrix is constructed for all such features identified in all the documents, which is then factorized to collect the topics. We used the identified words and searched the transcripts to identify and summarize the context of the discussions.

Youth from Lidingö expressed that a bad day would not define their mental health. They considered the persistence of a bad mood as poor mental health. They also indicated that individuals create a facade or hesitate to seek help. Routines were deemed essential to help with anxiety. It was difficult for them to differentiate between mental health and well-being.

Highly educated parents and household locations in Stockholm positively impact youth well-being. A recent study observed a similar trend [26]. Parental expectations of youth contribute negatively to youth well-being. In Sweden, the ninth-grade scores determine admissions to high schools and are considered essential.

Personnel from both municipalities stated that attending school is the most protective factor for youth. Therefore, many efforts focus on keeping youth in schools, reducing absenteeism, and returning youth to receive an education. They discussed the inclusion of individuals with disabilities, families with members who have mental illness, and teenage parents.

Safety issues were not discussed or observed in either municipality.

Expert participants observed that although much data have been collected, converting it into knowledge has been difficult. Many were keen to use data to inform their work, but have found it hard to find tools to collaborate across departments. Quantitative data were rarely enough to generate insights. They discussed the importance of events involving different departments in performing cross-sectoral analysis.

Expert participants also noted that negative aspects of mental health had been historically tracked, but positive effects of well-being promotion were not included in current surveys. Youths aged between 21 and 29 years were not adequately tracked to observe their transition into adulthood. The Stockholm survey, conducted nationally every 2 years with adolescents, was an example of a well-defined survey constructed from consultations across departments. Participants indicated that parameters such as free-time activities should be tracked regularly within surveys, for example, the Stockholm survey.

Experts from both municipalities identified that different departments track the youths’ free time. They indicated that while sports and outdoor activities improve youth well-being, parents sometimes need to participate in such activities. Such support could include transportation, organization, acquiring resources, and actively playing with them. This also allows parents to build relationships and trust across generations. Thus, free time available for parents also plays a role in the youths’ ability to access such programs. Nynäshamn personnel observed that attending organized sporting events becomes increasingly stressful as youth age. The stress of balancing studies and planning a career takes precedence over leisure activities.

The youth of Lidingö had more options to spend their free time than the youth in Nynäshamn. Girls suggested the development of leisure parks. They preferred more social activities compared to boys. The female participants indicated they would like to gather and do activities within leisure parks, expressing that they tend to participate less in organized sports. Participants noted that developing leisure parks for mixed use by both older adults and youth to engage in leisure activities would help multiple individuals within a municipality. However, football was popular among both boys and girls.

Expert participants of both municipalities identified that maintaining long-term collaborations across departments is a challenge. Nynäshamn created an interdepartmental group, such as collaboration between the children and educational administration and business-labor market administration, to improve labor access for their youth. Youths aged 19‐29 years who did not have a linear education path were provided support. A coordination agency that can direct various programs in Campus Nynäshamn has helped young people return to education. The program included financial assistance and education to enter the labor market. Programs such as Erasmus volunteer programs with visits to other countries have helped youth engage with studies and activities. Lidingö personnel also identified that summer jobs positively affected youth well-being. They allowed youth to gain professional experience and interact with people outside their social cliques.

## Discussion

### Principal Findings

In this work, we used data visualization as a collection of boundary objects in a structured workshop among participants to identify and understand the impact of programs on improving youth well-being in Lidingö and Nynäshamn municipalities of Sweden. We analyzed participatory workshop transcripts to identify the most critical topics discussed in all sessions. This enabled us to backtrack within the transcripts and identify the context for discussing the topic. We used the terms extracted to identify the sentences and contexts from the transcripts (Multimedia Appendix 7).

Visualizations provided the starting point for such discussions in each workshop. Almost all the participants observed that the visualizations helped them better situate their local datasets within the national statistics. Participants requested further fine-grained resolution for data visualizations and more combinations of visuals across departments for discussions. Participants in multiple sessions remarked that they could think of new ideas during these sessions.

The visualizations indeed acted as boundary objects. They allowed participants from different departments to trace individuals’ paths while pursuing their education and the social support they may access as they grow. They also allowed for a conversation on the different contexts in which an individual is situated, such as individuals who need social support or motivation to continue higher education.

Due to the existing administrative structure, personnel tend to focus on their local departments and goals. Any collected data and the eventual knowledge derived from the same end up in data silos within departments. This causes a myopic view of the municipal system. Our data visualizations included data from allied parts of the municipality. This gave participants insight into how their department is situated in the overall system and the work done by allied departments. They were also able to identify modifications to national surveys to track the transition from teenagers to adults better.

We saw evidence for “a boundary object effect” as participants could identify missing information from current data infrastructure and surveys, such as the ability to track individuals who turn 18 years of age and, thus, exit the youth programs. They may still need support after they achieve legal adulthood. This is currently not possible directly due to differences in data infrastructure and departments. We noted that this identification was possible due to visualizations connecting data from one department. Visualizing data from within a given department would not provide new insights for personnel from the same department.

### Limitations

Our current work used 15 workshops with participants from 2 municipalities. Increasing the number of sessions or extending dialogues would produce more extensive transcripts. Thus, the size and quality of the discussions were limitations. A trained facilitator can help produce better-quality dialogues.

We created visualizations based on themes surrounding youth mental well-being. During the workshops, some participants requested more on-the-fly visualizations. While this was achieved, a better tool that can quickly adapt to requests without technical complexity would allow for improved interactions between data and participants.

For the text analysis, we relied on the Natural Language Toolkit (NLTK) library’s stop-words for Swedish and our own set of identified Swedish stop-words. A better listing of stop-words would identify topics, avoiding common phrases (Multimedia Appendix 5).

### Conclusions

We observed that using fused data allowed us to create visualizations from a given department and data about related system parts that promote well-being. The visualizations allowed participants from different departments to relate to perspectives from associated departments, demonstrating that such contextualized visualizations could perform the function of a boundary object. We observed this in the interaction involving the sports department in the Nynäshamn municipality when participants identified that parents’ choice of sports and participation was necessary.

Both municipalities were able to contextualize well-being based on their local population demographics. Lidingö identified that the nature of stress the students feel relates to their academic performance and future aspirations. Nynäshamn identified that their youth need further motivation to continue their education and even aspire for a higher education. We observe here that although both municipalities have the same goal for achieving youth well-being when contextualized to their local situation, they present themselves as different challenges to the municipalities.

Participants from both municipalities (Lidingö and Nynäshamn) faced challenges with their youth populations, affecting their approach to promoting youth well-being. We observed that participants in both sessions requested new combinations for visualizations and dashboards, demonstrating variations in how they intend to use their data and knowledge.

Finally, both municipalities noted a need for changes to national surveys and data infrastructure collecting around the age group of 19‐25 years. Experts from educational departments noted that at this transition, their needs and support would stop being tracked by youth-based institutions. They will be considered adults and must approach adult (or general public) institutions for any support. This prevents experts from a better handoff to the adult system.

We plan to develop tools that enable municipal personnel to create visualizations on demand during sessions, directly using large language models and fused network databases. This would enhance interaction levels and promote interdepartmental collaboration. With further sessions with other Stockholm municipalities, we hope to see further nuances related to local effects.

## Supplementary material

10.2196/66377 Multimedia Appendix 1 Demonstrating visualizations of free time at Nynäshamn during a briefing session of a workshop.

10.2196/66377 Multimedia Appendix 2 List of all workshops conducted with participants and theme.

10.2196/66377 Multimedia Appendix 3 Swedish stop-words used for text analysis in addition to the Natural Language Toolkit.

10.2196/66377 Multimedia Appendix 4 Top words identified from intersections of all topics and workshops (Swedish).

10.2196/66377 Multimedia Appendix 5 Mapping for column names and file names for the matrix of occurrences of common topics extracted in all files.

10.2196/66377 Multimedia Appendix 6 List of common topics extracted from all the files and their occurrences in the lines of files.

10.2196/66377 Multimedia Appendix 7 Topic clusters identified from words in the transcripts of workshops with Lidingö.

10.2196/66377 Multimedia Appendix 8 Topic clusters identified from words in the transcripts of workshops with Nynäshamn.

10.2196/66377 Multimedia Appendix 9 Topic clusters identified from words in the transcripts of workshops with both municipalities.

## Abbreviations

NLTK: Natural Language Toolkit
OECD: Organisation of Economic Co-operation and Development
WHO: World Health Organization
