# Supplementary figures and images for: Issues in Identifying Strategies for Youth Mental Well-Being in Stockholm Municipalities Using Participatory Sessions and Text Mining: Qualitative Study
Source: Online J Public Health Inform. 2025 Jul 28;17:e66377. doi: 10.2196/66377 (PMC12303551; doi:10.2196/66377)

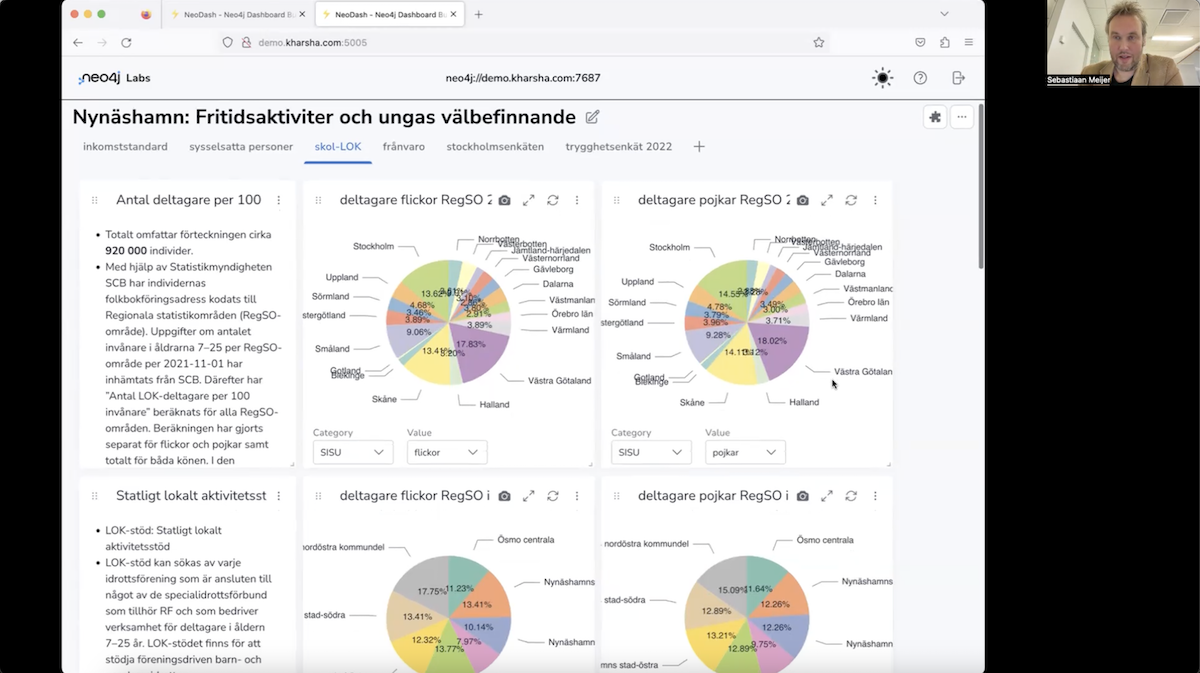

Supplement: Multimedia Appendix 1 [file ojphi-v17-e66377-s001.png]

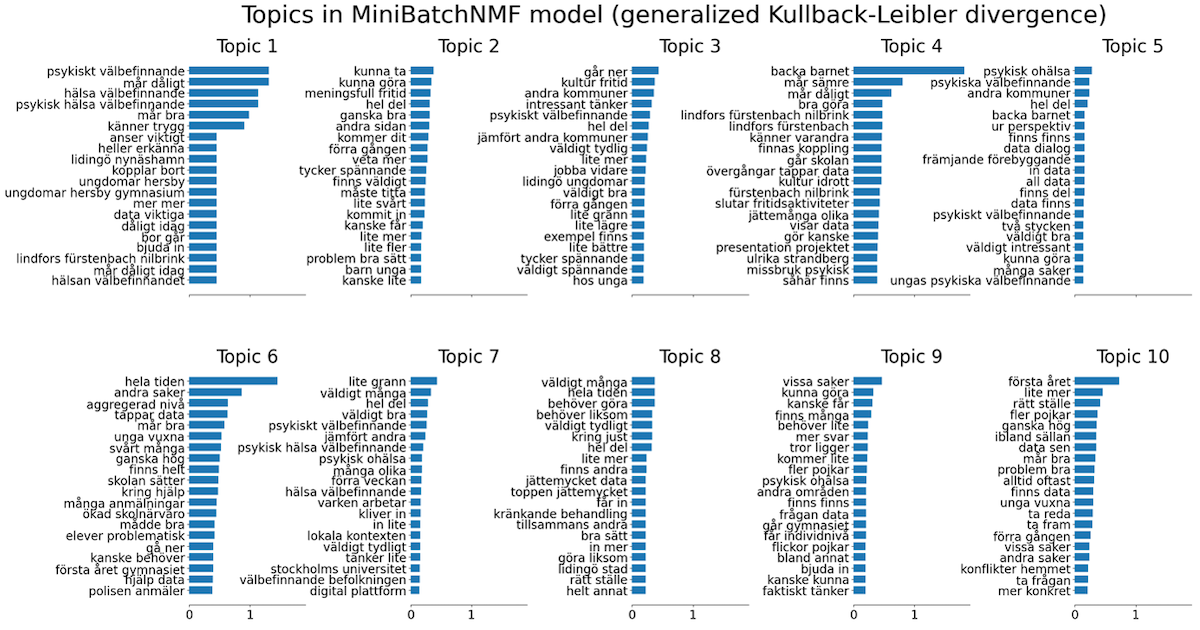

Supplement: Multimedia Appendix 7 [file ojphi-v17-e66377-s007.png]

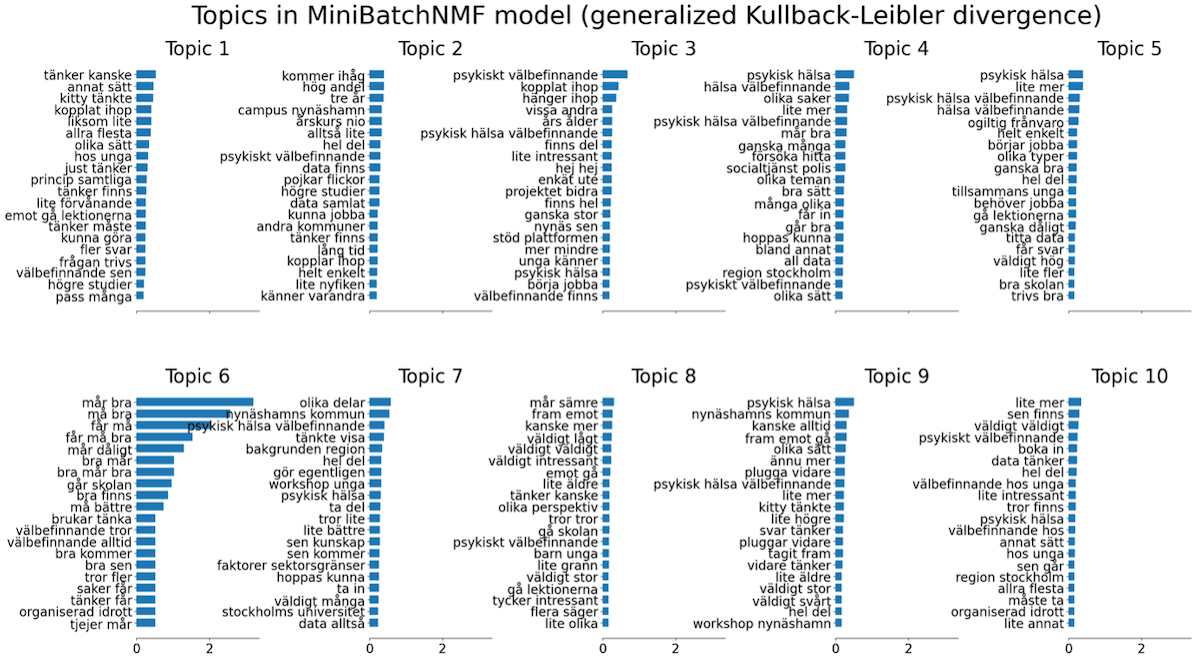

Supplement: Multimedia Appendix 8 [file ojphi-v17-e66377-s008.png]

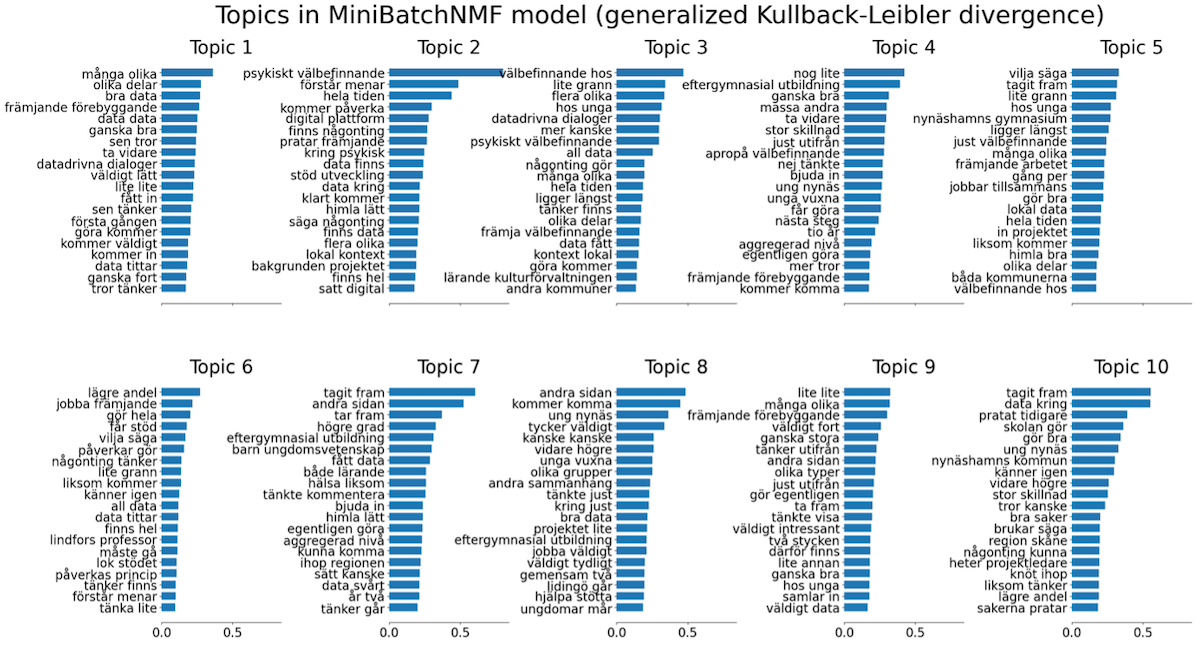

Supplement: Multimedia Appendix 9 [file ojphi-v17-e66377-s009.png]
