# Supplementary material for: Issues in Identifying Strategies for Youth Mental Well-Being in Stockholm Municipalities Using Participatory Sessions and Text Mining: Qualitative Study
Source: Online J Public Health Inform. 2025 Jul 28;17:e66377. doi: 10.2196/66377 (PMC12303551; doi:10.2196/66377)
Supplement: Multimedia Appendix 2 [file ojphi-v17-e66377-s002.docx]

| Date | Theme | Participants | Theme |
| --- | --- | --- | --- |
| 2/20/2023 | Workshop with young people | Lidingö | High school students from Hersby gymnasium |
| 4/4/2023 | First look at the data | Lidingö | Officials from Lidingö |
| 5/25/2023 | Meeting with policy makers | Lidingö | Officials from several different administrations |
| 8/14/2023 | Collection (those who missed) | Lidingö | Head of IT and Head of Care and Social Services |
| 9/4/2023 | Well-being of young people - data and reasoning | Both municipalities | Managers School and Social Administration, Lidingö Culture & Leisure - mixed |
| 9/11/2023 | First look at the data | Nynäshamn | Officials from Nynäshamn |
| 10/2/2023 | How do young people who do not continue their studies feel? | Nynäshamn | Officials from the upper secondary school, Campus Nynäshamn, social administration |
| 10/16/2023 | School+police+social services look at young people's wellbeing using data | Nynäshamn | Officials from schools, social services and neighbourhood police |
| 11/6/2023 | Leisure activities and mental well-being of young people | Nynäshamn | Head of Leisure Centres + representative of secondary schools |
| 11/9/2023 | Workshop with young people | Nynäshamn | Young people at the leisure centre in Ösmo |
| 11/13/2023 | School attendance and well-being | Nynäshamn | Schools, student health, social services |
| 11/20/2023 | The role of school health in promoting the mental well-being of young people | Both municipalities | Student Health Nynäshamn and Lidingö |
| 11/30/2023 | UVAs - Young people who are neither working nor studying | Lidingö | Social services, KAA (municipal activity responsibility), Delta ung, leisure centre, school psychologist |
| 12/7/2023 | Meaningful leisure | Lidingö | Representatives from leisure centres, libraries, the sports unit, general culture, etc. |
| 12/11/2023 | Finalisation of practical work in municipalities | Both municipalities | Managers School and Social Administration, Lidingö Culture & Leisure - mixed |
