# Supplementary material for: Issues in Identifying Strategies for Youth Mental Well-Being in Stockholm Municipalities Using Participatory Sessions and Text Mining: Qualitative Study
Source: Online J Public Health Inform. 2025 Jul 28;17:e66377. doi: 10.2196/66377 (PMC12303551; doi:10.2196/66377)
Supplement: Multimedia Appendix 3 [file ojphi-v17-e66377-s003.docx]

| och | harsha | 28 | 61 | 94 |
| --- | --- | --- | --- | --- |
| eller | krishna | 29 | 62 | 95 |
| inte | sebastiaan | 30 | 63 | 96 |
| att | sebastian | 31 | 64 | 97 |
| i | meijer | 32 | 65 | 98 |
| det | 0 | 33 | 66 | 99 |
| är | 1 | 34 | 67 |  |
| en | 2 | 35 | 68 |  |
| som | 3 | 36 | 69 |  |
| för | 4 | 37 | 70 |  |
| med | 5 | 38 | 71 |  |
| på | 6 | 39 | 72 |  |
| den | 7 | 40 | 73 |  |
| av | 8 | 41 | 74 |  |
| har | 9 | 42 | 75 |  |
| jag | 10 | 43 | 76 |  |
| var | 11 | 44 | 77 |  |
| men | 12 | 45 | 78 |  |
| om | 13 | 46 | 79 |  |
| så | 14 | 47 | 80 |  |
| tack | 15 | 48 | 81 |  |
| ar | 16 | 49 | 82 |  |
| också | 17 | 50 | 83 |  |
| ser | 18 | 51 | 84 |  |
| se | 19 | 52 | 85 |  |
| ska | 20 | 53 | 86 |  |
| ja | 21 | 54 | 87 |  |
| vill | 22 | 55 | 88 |  |
| vet | 23 | 56 | 89 |  |
| bara | 24 | 57 | 90 |  |
| fa | 25 | 58 | 91 |  |
| få | 26 | 59 | 92 |  |
| speaker | 27 | 60 | 93 |  |
