# Supplementary material for: Issues in Identifying Strategies for Youth Mental Well-Being in Stockholm Municipalities Using Participatory Sessions and Text Mining: Qualitative Study
Source: Online J Public Health Inform. 2025 Jul 28;17:e66377. doi: 10.2196/66377 (PMC12303551; doi:10.2196/66377)
Supplement: Multimedia Appendix 4 [file ojphi-v17-e66377-s004.docx]

| **Top Words** | |
| --- | --- |
| **Swedish** | **English** |
| andra kommuner | other municipalities |
| finns andra | there are others |
| förra veckan | last week |
| ganska stor | quite large |
| hela tiden | all the time |
| helt enkelt | quite simply |
| kanske lite | maybe a little |
| kanske mer | maybe more |
| lite mer | a little more |
| lite nyfiken | a little curious |
| lite olika | a little different |
| lite svårt | a little difficult |
| lång tid | long time |
| psykisk hälsa | mental health |
| ta fram | develop |
| unga vuxna | young adults |
| väldigt bra | very good |
| väldigt viktigt | very important |
| väldigt väldigt | very very |
| andra länder | other countries |
| andra sidan | other side |
| annat sätt | other way |
| avdelningschef stöd | head of department support |
| barn unga | children young people |
| behöver hjälp | need help |
| behöver jobba | need to work |
| bra göra | good do |
| bra kommer | good will |
| börja jobba | start working |
| data kring | data about |
| finns del | exists part |
| finns finns | exists exists |
| finns hel | exists whole |
| finns hel del | there is a lot |
| finns många | there are many |
| flickor pojkar | girls boys |
| får svar | receive answers |
| fått in | received in |
| försöker hitta | trying to find |
| ganska bra | quite well |
| ganska fort | quite fast |
| ganska hög | quite high |
| gå in | going in |
| går bra | going well |
| går gymnasiet | going to secondary school |
| går skolan | going to school |
| gått gymnasiet | go to high school |
| gör får | doing sheep |
| gör lite | do a little |
| göra någonting | do something |
| hel del | a lot |
| helt annat | completely different |
| in liksom | in like |
| intressant tänker | interesting thinking |
| kanske kunna | might be able to |
| kommer in | come in |
| kultur fritid | culture leisure |
| kunna göra | be able to do |
| kunna komma | be able to come |
| känner varandra | know each other |
| liksom lite | a little bit |
| lite grann | a little bit |
| lite lite | a little bit |
| lägre andel | lower proportion |
| många olika | many different |
| mår bra | feel good |
| mår dåligt | feel bad |
| nynäshamns gymnasium | nynäshamn upper secondary school |
| olika grupper | different groups |
| olika sätt | different ways |
| olika typer | different types |
| precis säger | just say |
| psykisk ohälsa | mental illness |
| scb data | scb data |
| sen kanske | then maybe |
| sitt liv | his life |
| stor roll | major role |
| stöd utveckling | support development |
| ta in | take in |
| tror kommer | think will |
| tänker kanske | think maybe |
| tänker kommer | think will |
| väl kanske | well maybe |
| väldigt många | very many |
| väldigt svårt | very difficult |
| väldigt tydligt | very clear |
| ännu mer | even more |
| aggregerad nivå | aggregated level |
| flera olika | several different |
| flera år | several years |
| främjande förebyggande | promotion prevention |
| gör kanske | may do |
| göra finns | do exist |
| jobba vidare | work on |
| kanske behöver | may need |
| kanske finns | may be available |
| nästa steg | next step |
| någonting annat | something else |
| olika delar | different parts |
| sen sen | then then |
| ta reda | find out |
| ta vidare | take further |
| tror tänker | think thinks |
| tänker tänker | think think |
