# Supplementary material for: Issues in Identifying Strategies for Youth Mental Well-Being in Stockholm Municipalities Using Participatory Sessions and Text Mining: Qualitative Study
Source: Online J Public Health Inform. 2025 Jul 28;17:e66377. doi: 10.2196/66377 (PMC12303551; doi:10.2196/66377)
Supplement: Multimedia Appendix 5 [file ojphi-v17-e66377-s005.docx]

| **Column Name** | **File Name** |
| --- | --- |
| N1 | 2023_11_06_leisure_nynas.txt |
| N2 | 2023_10_02_young_unemployed_nynas.txt |
| N3 | 2023_09_11_firstLook_nynas.txt |
| N4 | 2023_11_13_attendance_nynas.txt |
| N5 | 2023_11_09_students_osmo_nynas.txt |
| N6 | 2023_10_16_school_police_nynas.txt |
| L1 | 2023_04_04_datadiscussion_lidingo.txt |
| L2 | 2023_12_07_freetime_lidingo.txt |
| L3 | 2023_02_02_youth_hesby_lidingo.txt |
| L4 | 2023_08_14_wrap_session_lidingo.txt |
| L5 | 2023_11_30_youth_notworking_lidingo.txt |
| L6 | 2023_05_25_decisionmakers_lidingo.txt |
| J1 | 2023_09_04_schools_joint.txt |
| J2 | 2023_12_11_finalisation.txt |
| J3 | 2023_09_04_data_joint.txt |
