# Supplementary material for: Issues in Identifying Strategies for Youth Mental Well-Being in Stockholm Municipalities Using Participatory Sessions and Text Mining: Qualitative Study
Source: Online J Public Health Inform. 2025 Jul 28;17:e66377. doi: 10.2196/66377 (PMC12303551; doi:10.2196/66377)
Supplement: Multimedia Appendix 6 [file ojphi-v17-e66377-s006.docx]

| **Word Swedish** | **Word English** | **N1** | **N2** | **N3** | **N4** | **N5** | **N6** | **L1** | **L2** | **L3** | **L4** | **L5** | **L6** | **J1** | **J2** | **J3** |
| --- | --- | --- | --- | --- | --- | --- | --- | --- | --- | --- | --- | --- | --- | --- | --- | --- |
| olika sätt | different ways | 0 | 0 | 120.13 | 145.183 | 0 | 18,30,94,102 | 0 | 156 | 0 | 45 | 28 | 0 | 0 | 0 | 0 |
| psykisk ohälsa | mental illness | 153 | 4.44 | 26,68,72 | 0 | 31.34 | 10,22,66,86,94,110 | 0 | 0 | 22 | 45 | 28,324,346,348,444 | 12.48 | 0 | 0 | 68 |
| lite mer | a little more | 41,249,257,319,325 | 44 | 26,36,158 | 183 | 0 | 10,110,130 | 0 | 130 | 0 | 61,73,77 | 54.384 | 42,168,274,380 | 0 | 171.329 | 106,146,224 |
| kunna göra | be able to do | 97 | 128 | 116 | 211.221 | 0 | 0 | 0 | 0 | 0 | 0 | 100,234,380,388 | 290,454,464,604 | 0 | 41,119,303 | 0 |
| andra länder | other countries | 0 | 240 | 0 | 187 | 0 | 0 | 0 | 50 | 0 | 9 | 112 | 0 | 0 | 0 | 0 |
| psykisk hälsa | mental health | 53.57 | 0 | 2,26,52 | 0 | 0 | 62.122 | 76 | 0 | 12,18,20,26,44,84,94 | 21,27,45,87 | 112,288,450 | 86 | 0 | 203 | 94.234 |
| många olika | many different | 0 | 0 | 130 | 0 | 0 | 66 | 132 | 86 | 0 | 61 | 160 | 0 | 0 | 193 | 0 |
| olika typer | different types | 0 | 0 | 0 | 161 | 0 | 58 | 0 | 158 | 0 | 0 | 160 | 0 | 0 | 0 | 94 |
| väldigt bra | very good | 0 | 4 | 0 | 177 | 0 | 0 | 0 | 46,62,130 | 0 | 49 | 160,386,398 | 0 | 148 | 177,275,355,371 | 0 |
| data kring | data about | 41 | 44 | 0 | 0 | 0 | 0 | 0 | 0 | 0 | 37.79 | 208.318 | 0 | 0 | 0 | 144 |
| andra kommuner | other municipalities | 41 | 114,120,238 | 0 | 0 | 0 | 10 | 0 | 50.132 | 0 | 0 | 224,234,286,288 | 162 | 72.74 | 41 | 0 |
| börja jobba | start working | 57 | 182 | 0 | 0 | 0 | 0 | 0 | 0 | 0 | 51 | 224 | 0 | 0 | 0 | 208 |
| ganska fort | quite fast | 107 | 44 | 26 | 0 | 0 | 0 | 0 | 0 | 0 | 13.71 | 224 | 0 | 0 | 0 | 0 |
| ganska stor | quite large | 41 | 0 | 0 | 109 | 0 | 10 | 0 | 70 | 0 | 31.35 | 224 | 12 | 0 | 41,203,399 | 208 |
| hel del | a lot | 41.65 | 44 | 26.54 | 0 | 0 | 10 | 0 | 0 | 0 | 15,21,47,49 | 224,494,498 | 2,320,604 | 0 | 0 | 106 |
| ta in | take in | 219 | 0 | 26,36,52,164 | 0 | 0 | 10 | 127 | 62 | 0 | 61 | 224,238,278,366 | 68.294 | 0 | 315 | 208.296 |
| väldigt många | very many | 0 | 44.252 | 130 | 0 | 0 | 30 | 0 | 52.88 | 0 | 5,13,23,43 | 262.402 | 0 | 0 | 193 | 0 |
| olika delar | different parts | 0 | 0 | 26.112 | 0 | 0 | 0 | 0 | 46 | 0 | 61 | 278 | 334 | 178 | 181.295 | 90 |
| annat sätt | other way | 309.317 | 0 | 36 | 165.211 | 0 | 94,106,114 | 0 | 0 | 0 | 65.71 | 288,418,422 | 398 | 0 | 0 | 302.326 |
| kanske finns | may be available | 0 | 0 | 0 | 0 | 0 | 0 | 0 | 0 | 0 | 49 | 288 | 0 | 0 | 0 | 86.296 |
| förra veckan | last week | 0 | 4 | 0 | 0 | 0 | 114 | 0 | 0 | 0 | 0 | 320 | 0 | 0 | 171 | 214 |
| kommer in | come in | 0 | 44,92,210,236 | 0 | 117 | 8 | 0 | 0 | 50,52,74 | 0 | 0 | 320,384,482 | 444.546 | 84 | 0 | 94 |
| lång tid | long time | 0 | 92,96,236 | 12 | 183.187 | 0 | 0 | 0 | 70 | 0 | 0 | 320 | 280 | 0 | 363 | 94.208 |
| lite olika | a little different | 0 | 0 | 26.44 | 0 | 0 | 10 | 0 | 120 | 0 | 0 | 338 | 2.376 | 0 | 63.299 | 98.158 |
| ta reda | find out | 0 | 0 | 0 | 0 | 0 | 0 | 0 | 0 | 0 | 0 | 352 | 294,462,464 | 0 | 347 | 80 |
| lite svårt | a little difficult | 0 | 60 | 26 | 0 | 0 | 10 | 0 | 32 | 0 | 0 | 356 | 2.258 | 0 | 0 | 82 |
| gå in | going in | 0 | 0 | 124 | 173 | 0 | 42 | 0 | 0 | 0 | 0 | 366.398 | 162 | 0 | 0 | 158 |
| lite grann | a little bit | 0 | 48 | 12 | 0 | 0 | 22.94 | 0 | 30,48,66,84,154,156 | 0 | 0 | 366 | 0 | 0 | 233 | 0 |
| hela tiden | all the time | 41 | 156 | 0 | 173 | 0 | 30.74 | 91,92,143 | 0 | 0 | 53,55,61 | 370,374,494 | 0 | 120,144,148,170 | 177 | 0 |
| kanske kunna | might be able to | 0 | 0 | 0 | 0 | 0 | 0 | 0 | 0 | 0 | 0 | 384 | 0 | 158 | 0 | 0 |
| kanske lite | maybe a little | 0 | 0 | 26 | 0 | 0 | 122 | 0 | 52 | 0 | 0 | 384 | 0 | 0 | 0 | 0 |
| flera år | several years | 0 | 88.9 | 0 | 0 | 0 | 0 | 0 | 0 | 0 | 0 | 438,444,498 | 258 | 0 | 119 | 242 |
| göra någonting | do something | 0 | 186 | 52 | 0 | 0 | 0 | 0 | 46 | 0 | 0 | 456.494 | 0 | 0 | 0 | 0 |
| någonting annat | something else | 0 | 102 | 0 | 0 | 0 | 0 | 0 | 40 | 0 | 0 | 456 | 210 | 92 | 0 | 234 |
| sitt liv | his life | 0 | 44 | 218 | 0 | 0 | 114 | 0 | 66 | 0 | 0 | 460 | 0 | 0 | 0 | 0 |
| helt annat | completely different | 0 | 102 | 0 | 0 | 0 | 94 | 0 | 0 | 0 | 65.71 | 492 | 0 | 0 | 0 | 0 |
| behöver hjälp | need help | 0 | 0 | 218 | 161 | 0 | 70 | 0 | 60 | 0 | 0 | 494 | 0 | 0 | 0 | 0 |
| ta vidare | take further | 0 | 0 | 0 | 0 | 0 | 0 | 9 | 158 | 0 | 0 | 0 | 0 | 0 | 221 | 0 |
| ganska hög | quite high | 0 | 0 | 26 | 25 | 0 | 0 | 29 | 0 | 0 | 0 | 0 | 44.142 | 0 | 0 | 0 |
| aggregerad nivå | aggregated level | 0 | 0 | 0 | 0 | 0 | 0 | 70 | 0 | 0 | 61 | 0 | 0 | 0 | 59 | 204 |
| främjande förebyggande | promotion prevention | 0 | 0 | 0 | 0 | 0 | 0 | 77 | 0 | 0 | 0 | 0 | 0 | 0 | 61,177,203 | 0 |
| mår dåligt | feel bad | 0 | 0 | 0 | 0 | 34,37,55 | 78 | 95 | 0 | 73,78,115 | 0 | 0 | 322 | 0 | 0 | 0 |
| kanske behöver | may need | 0 | 0 | 0 | 0 | 0 | 10 | 119 | 82 | 0 | 0 | 0 | 0 | 120 | 0 | 0 |
| helt enkelt | quite simply | 9.193 | 44,96,144,182 | 68.158 | 47,63,143 | 0 | 42.62 | 133 | 36,40,60 | 0 | 67 | 0 | 294 | 170 | 0 | 90 |
| fått in | received in | 0 | 0 | 12 | 3 | 0 | 0 | 0 | 0 | 0 | 13 | 0 | 68 | 0 | 0 | 316 |
| ganska bra | quite well | 41 | 0 | 0 | 0 | 0 | 10 | 0 | 0 | 0 | 13 | 0 | 520 | 0 | 0 | 0 |
| ännu mer | even more | 193 | 0 | 26 | 195 | 0 | 10,106,110 | 0 | 0 | 0 | 21 | 0 | 608 | 0 | 171.181 | 0 |
| väldigt tydligt | very clear | 0 | 48 | 0 | 161 | 0 | 0 | 0 | 20 | 0 | 23,45,87 | 0 | 0 | 0 | 0 | 144.288 |
| väl kanske | well maybe | 0 | 244 | 0 | 0 | 0 | 0 | 0 | 0 | 0 | 29 | 0 | 564 | 0 | 197 | 0 |
| nästa steg | next step | 0 | 0 | 0 | 0 | 0 | 0 | 0 | 50 | 0 | 37 | 0 | 0 | 0 | 113 | 214.224 |
| mår bra | feel good | 0 | 0 | 52.54 | 173 | 8,22,33,52,53 | 78 | 0 | 0 | 49,77,115 | 45 | 0 | 0 | 0 | 0 | 0 |
| finns andra | there are others | 0 | 156 | 0 | 0 | 0 | 0 | 0 | 0 | 0 | 49.71 | 0 | 248 | 124 | 299 | 0 |
| försöker hitta | trying to find | 0 | 0 | 0 | 0 | 0 | 0 | 0 | 0 | 0 | 51 | 0 | 518 | 0 | 0 | 0 |
| liksom lite | like a little bit | 213 | 0 | 0 | 0 | 0 | 0 | 0 | 30 | 0 | 61.89 | 0 | 0 | 0 | 0 | 0 |
| unga vuxna | young adults | 219.319 | 136 | 108 | 187 | 0 | 0 | 0 | 0 | 0 | 61 | 0 | 22.364 | 0 | 113 | 0 |
| flera olika | several different | 0 | 0 | 0 | 0 | 0 | 0 | 0 | 0 | 0 | 61 | 0 | 0 | 148 | 111 | 0 |
| ta fram | develop | 0 | 0 | 112 | 39 | 0 | 74 | 0 | 70 | 0 | 89 | 0 | 142.376 | 0 | 119.347 | 86 |
| väldigt viktigt | very important | 291 | 0 | 0 | 0 | 0 | 118 | 0 | 36.156 | 116 | 0 | 0 | 0 | 166 | 0 | 82 |
| jobba vidare | work on | 0 | 0 | 0 | 0 | 0 | 0 | 0 | 36.112 | 0 | 0 | 0 | 564 | 0 | 175.371 | 208 |
| stor roll | major role | 0 | 200 | 0 | 0 | 0 | 126 | 0 | 50 | 0 | 0 | 0 | 464 | 0 | 0 | 0 |
| får svar | receive answers | 0 | 0 | 0 | 55,63,149,221 | 0 | 66 | 0 | 54 | 0 | 0 | 0 | 0 | 0 | 0 | 0 |
| lite nyfiken | a little curious | 0 | 64.226 | 60 | 0 | 0 | 0 | 0 | 70 | 0 | 0 | 0 | 248 | 0 | 389 | 258 |
| andra sidan | other side | 0 | 142 | 0 | 0 | 0 | 118 | 0 | 158 | 0 | 0 | 0 | 136.524 | 0 | 203 | 0 |
| finns många | there are many | 0 | 0 | 78.164 | 0 | 0 | 0 | 0 | 0 | 0 | 0 | 0 | 64 | 0 | 0 | 0 |
| behöver jobba | need to work | 0 | 0 | 0 | 169 | 0 | 10.62 | 0 | 0 | 0 | 0 | 0 | 232 | 0 | 0 | 0 |
| känner varandra | know each other | 0 | 18.128 | 0 | 0 | 51 | 0 | 0 | 0 | 0 | 0 | 0 | 334 | 0 | 0 | 0 |
| gör lite | do a little | 0 | 0 | 26 | 0 | 0 | 0 | 0 | 0 | 0 | 0 | 0 | 0 | 0 | 0 | 0 |
| väldigt svårt | very difficult | 0 | 78 | 54 | 143.145 | 0 | 46.74 | 0 | 0 | 0 | 0 | 0 | 0 | 56.92 | 0 | 142 |
| kunna komma | be able to come | 107 | 0 | 140 | 79.169 | 0 | 26.54 | 0 | 0 | 0 | 0 | 0 | 0 | 178 | 0 | 326 |
| olika grupper | different groups | 0 | 44 | 158 | 0 | 0 | 0 | 0 | 0 | 0 | 0 | 0 | 0 | 0 | 193 | 94 |
| kanske mer | maybe more | 0 | 0 | 0 | 79.135 | 0 | 10,34,94 | 0 | 0 | 0 | 0 | 0 | 0 | 0 | 0 | 112.288 |
| bra kommer | good will | 0 | 0 | 0 | 0 | 8 | 0 | 0 | 0 | 0 | 0 | 0 | 0 | 0 | 0 | 0 |
| sen kanske | then maybe | 0 | 0 | 0 | 0 | 0 | 22 | 0 | 0 | 0 | 0 | 0 | 0 | 0 | 0 | 0 |
| lite lite | a little bit | 0 | 60 | 0 | 0 | 0 | 70 | 0 | 0 | 0 | 0 | 0 | 0 | 0 | 0 | 0 |
| flickor pojkar | Girls, boys | 41 | 0 | 0 | 0 | 0 | 0 | 0 | 0 | 0 | 0 | 0 | 0 | 0 | 0 | 0 |
| lägre andel | lower proportion | 41 | 158 | 0 | 0 | 0 | 0 | 0 | 0 | 0 | 0 | 0 | 0 | 0 | 0 | 214 |
